# A novel diagnostic model for insulinoma

**DOI:** 10.1007/s12672-022-00534-w

**Published:** 2022-08-02

**Authors:** Feng Wang, Zhe Yang, XiuBing Chen, Yiling Peng, HaiXing Jiang, ShanYu Qin

**Affiliations:** Department of Gastroenterology, The First Affiliated Hospital of Guangxi Medical University, No. 6 Shuangyong Road, Nanning, 530021 Guangxi Zhuang Autonomous Region People’s Republic of China

**Keywords:** Insulinoma, Hypoglycemia, Diagnostic predictive model

## Abstract

The aim is to describe a simple and feasible model for the diagnosis of insulinoma. This retrospective study enrolled 37 patients with insulinoma and 44 patients with hypoglycemia not due to insulinoma at the First Affiliated Hospital of Guangxi Medical University. General demographic and clinical characteristics; hemoglobin A1c (HbA1c), insulin and C-peptide concentrations; and the results of 2-h oral glucose tolerance tests (OGTT) were recorded, and a logistic regression model predictive of insulinoma was determined. Body mass index (BMI), HbA1c concentration, 0-h C-peptide concentration, and 0-h and 1-h plasma glucose concentrations (***P*** < 0.05 each) were independently associated with insulinoma. A regression prediction model was established through multivariate logistics regression analysis: Logit p = 7.399+(0.310 × BMI) − (1.851 × HbA1c) − (1.467 × 0-h plasma glucose) + (1.963 × 0-h C-peptide) − (0.612 × 1-h plasma glucose). Using this index to draw a receiver operating characteristic (ROC) curve, the area under the curve (AUC) was found to be 0.957. The optimal cut-off value was − 0.17, which had a sensitivity of 89.2% and a specificity of 86.4%. Logit P ≥ − 0.17 can be used as a diagnostic marker for predicting insulinoma in patients with hypoglycemia.

## Introduction

Insulinoma is one of the most common functional pancreatic neuroendocrine tumors, originating from pancreatic β cells [1]. Its clinical manifestations include hyperinsulinemia with hypoglycemia syndrome [2]. The incidence of insulinoma in the general population has been estimated to be 1–4 per million people, but this may be an underestimate, as insulinomas have been found in 0.8–10% of cases in autopsy reports [3]. Insulinoma is often misdiagnosed as epilepsy, psychosis, or cerebrovascular disease [4–6]. More than half of patients require at least 3.6 years to be properly diagnosed, with many patients never being diagnosed correctly. Brain damage due to persistent hypoglycemia is irreversible and can lead to neurological sequelae. Therefore, early diagnosis and treatment are essential for good patient prognosis [7].

At present, the 72-h fasting test is the standard method of diagnosing insulinoma. About 80% of patients can be diagnosed within 24 h, and 100% can be diagnosed within 72 h [8]. Many patients, however, refuse to participate in 72-h fasting tests because they were unable to tolerate the distress caused by hunger, thus hindering the diagnosis of insulinoma [9]. Insulinoma can also be diagnosed by administration of the GLP1 peptide analogue exendin-4, followed by single-photon emission computed tomography (SPECT) [10]. Although this assay has a sensitivity of 95%, the need for costly equipment and inspections has limited the application of this method to the diagnosis of insulinoma. A simple and convenient diagnostic method is therefore needed.

By consulting the literature, patients with insulinoma will have changes in plasma glucose, C-peptide, and insulin due to the massive secretion of insulin [11–14], and 72% of patients will gain weight [15], we selected indicators related to the assessment of insulin, including insulin, plasma glucose, C-peptide, and HbA1c concentrations, as well as patient demographic and clinical characteristics. These indicators were utilized to establish a diagnostic model based on single factor analysis and multi-factor logistic regression analysis. Fajans’ and Turner’s indices are indicators of endogenous hyperinsulinemia [16], and our results show that our model outperforms these indices.

## Materials and methods

### Patient selection

**Fig. 1:**
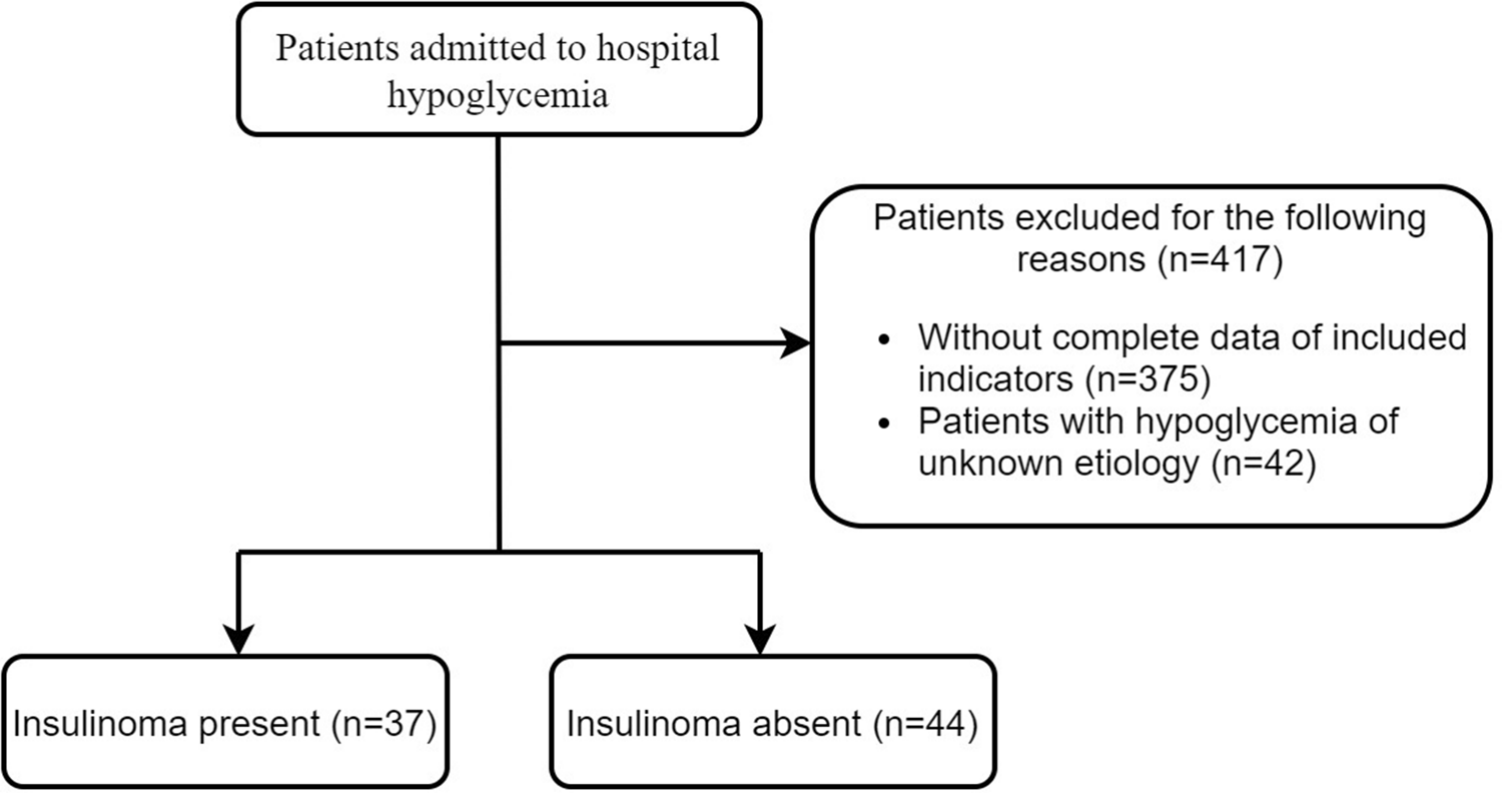
Flowchart of the sample selection

This study included 81 patients with hypoglycemia who were admitted to the First Affiliated Hospital of Guangxi Medical University from January 2012 to September 2021, including 37 patients with insulinoma and 44 with hypoglycemia of other etiologies. Insulinoma was confirmed by pathological examination of tissue samples following surgical resection or following ultrasound-guided fine needle endoscopic aspiration biopsy. Patients were included in the insulinoma group if they had hypoglycemia with Whipple’s triad (hypoglycemic manifestation, plasma glucose level < 2.8 mmol/l, and improvement in the symptoms after taking in glucose) and positive results (plasma glucose levels ≤ 40 mg/dl, insulin ≥ 36 pmol/l, C-peptide ≥ 200 pmol/l, proinsulin ≥ 5 pmol/l, β-hydroxybutyrate ≤ 2.7 mmol/l and absence of plasma or urine sulfonylurea metabolites) on the 72-h fasting test. Patients were included in the control group if they had hypoglycemia not due to insulinoma with Whipple’s triad and negative results on the 72-h fasting test, but due to other causes, such as hyperthyroidism, subtotal gastrectomy, severe liver disease, etc. Patients with incomplete clinical data were excluded (Fig. 1). The study protocol was approved by the Ethics Committee of the First Affiliated Hospital of Guangxi Medical University.

### Pathological examination of tissue specimens

Immediately after isolation, specimens were fixed in 10% formaldehyde solution and stained with hematoxylin-eosin. All samples were evaluated by two experienced pathologists.

### Clinical and laboratory index testing

Age, sex, height, and weight were recorded for each patient, and body mass index (BMI) was calculated as kg/m2. HbA1c concentrations were measured, and all patients underwent 75 g OGTT, as recommended by the World Health Organization. Venous blood samples were obtained directly before (0 min) and during the OGTT (30, 60, and 120 min). Glucose, C-peptide, and insulin concentrations were measured at 0, 1 and 2 h. Plasma glucose concentration was measured by the hexokinase method on a Cobase e702 analyzer (Roche, Germany). C-peptide and insulin concentrations were measured by electrochemiluminescence (Shanghai Enzymelink Biotechnology Corp.), according to the manufacturer’s instructions; and HbA1c concentrations were measured using a glycated hemoglobin instrument (BioRad Variant II).

### Statistical analyses

Normally distributed continuous variables were reported as mean ± standard deviation and compared by independent sample t tests. Non-normally distributed continuous variables were reported as median [first quartile, third quartile] and compared by independent sample rank sum tests. Categorical variables were reported as number (%) and compared by chi-square tests. Multivariate stepwise logistic regression analysis was used to evaluate the diagnostic value of the developed model, with receiver operating characteristic (ROC) curves delimiting the value and areas under the curve (AUC) compared using normal Z tests. All statistical analyses were performed using SPSS 24.0 software, with ***P*** < 0.05 considered statistically significant.

## Results

### Characteristics of patients in the Insulinoma and Control Groups

The demographic and clinical characteristics of the 81 patients with hypoglycemia, including 37 with and 44 without insulinoma, are shown in Table 1. Age, sex 1-h C-peptide, 1-h insulin, 2-h insulin and 2-h C-peptide concentrations in these two groups did not differ significantly (***P*** > 0.05). In contrast, BMI, 0-h C-peptide and 0-h insulin were significantly higher, and HbA1c, 0-h plasma glucose, 1-h and 2-h plasma glucose concentrations were significantly lower in the insulinoma than in the control group. Of the 37 insulinoma patients, 6 (16.2%), 27 (73.0%), and 4 (10.8%) had tumors of maximum diameters < 1 cm, 1–2 cm, and > 2 cm, respectively. Of these 37 tumors, 13 (35.1%) were located in the head, 11 (29.7%) in the body, and 13 (35.1%) in the tail of the pancreas. Thirty-six (97.3%) insulinoma were single and one (2.7%) was multiple. Of the 44 patients in the control group, 15 (34.1%) had preprandial hypoglycemia, 28 (63.6%) had of postprandial hypoglycemia, and one (2.3%) had induced hypoglycemia.

**Table 1:** Baseline demographic and clinical characteristics of patients in the insulinoma and control groups

Index	Insulinoma group (n = 37)	Control group (n = 44)		t/z/χ^2^	p
Age (years)	48.00 (37.50, 53.00)	50.00 (36.50, 76.00)		1.546	0.122
Sex(male/female)	14/23	25/19		2.900	0.089
BMI (kg/m^2^)	25.61 ± 4.52	22.54 ± 3.68		3.373	**0.001**
HbA1c (%)	4.71 ± 0.44	5.44 ± 0.66		5.705	**< 0.001**
0-h plasma glucose (mmol/L)	2.87 ± 1.07	4.15 ± 0.90		5.856	**< 0.001**
0-h C-peptide (ng/ml)	2.79 (1.88, 3.68)	1.79 (1.16, 2.81)		3.110	**0.002**
0-h insulin (pmol/l)	92.98 (52.17, 146.68)	30.65 (21.29, 54.25)		4.191	**< 0.001**
1-h plasma glucose (mmol/L)	6.47 ± 2.19	8.33 ± 2.70		3.365	**0.001**
1-h C-peptide (ng/ml)	6.76 ± 3.75	8.42 ± 4.15		1.866	0.066
1-h insulin (pmol/l)	385.10 (244.35, 562.45)	362.15 (198.46, 772.18)		0.057	0.955
2-h plasma glucose (mmol/L)	5.89 ± 2.30	7.07 ± 2.87		2.022	**0.047**
2-h C-peptide (ng/ml)	6.65 (4.14, 8.04)	7.52 (4.92, 10.64)		1.531	0.126
2-h insulin (pmol/l)	384.30 (191.90, 642.95)	285.26 (152.80, 456.95)		1.071	0.284

Data are mean ± SD or median [Q1,Q3]

BMI, body mass index; HbA1c, hemoglobin A1c

P values represent between-group comparisons

Bold indicates significant results with *P* < 0.05

### Construction of a diagnostic model for insulinoma

Single factor analysis yielded seven meaningful indicators, including BMI, and HbA1c, 0-h C-peptide, 0-h insulin and 0-h, 1-h and 2-h plasma glucose concentrations (***P*** < 0.05 each; Table 1). Multiple logistic regression analysis identified five factors significantly and independently associated with the occurrence of insulinoma, including higher BMI and 0-h C-peptide concentration and lower HbA1c, 0-h plasma glucose, and 1-h plasma glucose concentrations (***P*** < 0.05 each; Table 2). These five factors were incorporated into a regression prediction model, calculated using the formula: Logit P = 7.399 + ( 0.310 × BMI) − (1.851 × HbA1c) − (1.467 × 0-h plasma glucose) + (1.963 × 0-h C-peptide) − (0.612 × 1-h plasma glucose).

**Table 2:** Multiple logistic regression analysis of factors affecting the occurrence of insulinoma

Index	B	SE	Wals	P	OR	95%OR	
						Lower limit	Upper limit
BMI (kg/m^2^)	0.310	0.140	4.905	0.027	1.363	1.036	1.793
HbA1c (%)	− 1.851	0.830	4.967	0.026	0.157	0.031	0.800
0-h plasma glucose (mmol/L)	− 1.467	0.568	6.679	0.010	0.231	0.076	0.702
0-h C-peptide (ng/ml)	1.963	0.787	6.226	0.013	7.124	1.524	33.308
0-h insulin (pmol/l)	− 0.001	0.009	0.010	0.922	0.999	0.981	1.018
1-h plasma glucose (mmol/L)	− 0.612	0.255	5.742	0.017	0.542	0.329	0.895
2-h plasma glucose (mmol/L)	− 0.026	0.222	0.014	0.906	0.974	0.630	1.506
Constant	7.399	4.483	2.724	0.099	1634.863		

BMI, body mass index; HbA1c, hemoglobin A1c; B, Partial regression coefficient value; SE, Standard error; Wals, Wald chi-square value; P, probability; OR, odds ratio

### Predictive value of Logit P, Fajans’ index and Turner’s index for the diagnosis of insulinoma

**Fig. 2:**
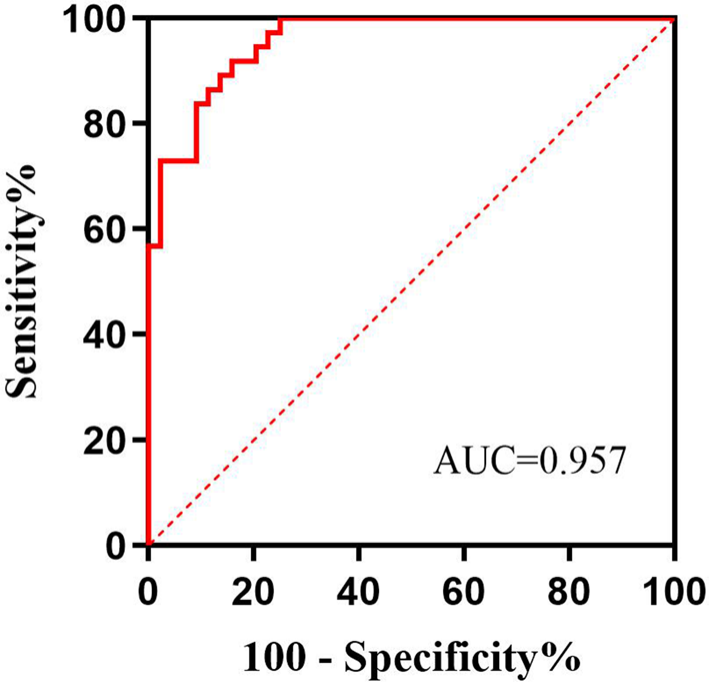
Diagnostic efficacy of the Logit P model, Fajans’ index, Turner’s index and verification of two models. Fajans’ index = immunoreactive insulin/glucose; Turner’s index = insulin * 100/(glucose − 30). Liao’s model = 8.305–0.441 * insulin 2 h/0 h ratio − 1.679 * C-peptide 1 h/0 h ratio. FPG*HBA1C index = FPG*HBA1C. PPV, positive predictive value; NPV, negative predictive value; AUC, area under the curve; CI, confidence interval. ^#^: Validated model

ROC curve analysis was used to compare the diagnostic performances of the Logit P, Fajans’ index and Turner’s index for the diagnosis of insulinoma. The Logit P insulinoma diagnostic model described in this study was found to have a higher accuracy rate, than either Fajans’ or Turner’s index (Table 3; Fig. 2).

**Table 3:** Diagnostic efficacy of the Logit P model, Fajans’ index, Turner’s index and verification of two models

Project	Cut off		Sensitivity (%)		Specificity (%)	PPV (%)		NPV (%)		Accuracy (%)		AUC (95%CI)		*P*	
Logit P	− 0.17		89.2		86.4	84.6		90.5		87.7		0.957(0.920–0.994)		0.001	
Fajans’ index	0.77		81.1		77.3	75.0		82.9		79.0		0.835(0.745–0.926)		0.001	
Turner’s index	261.69		59.5		90.9	84.6		72.7		76.5		0.746(0.624–0.867)		0.001	
^a^Liao’s model ^[9]^	0.351		70.3		65.9	70.3		65.9		67.9		–		–	
^a^FPG*HBA1C index ^[34]^	447.1		100		36.4	56.9		100		65.4		–		–	

Fajans’ index = immunoreactive insulin/glucose; Turner’s index = insulin * 100/(glucose − 30). Liao’s model = 8.305–0.441 * insulin 2 h/0 h ratio − 1.679 * C-peptide 1 h/0 h ratio. FPG*HBA1C index = FPG*HBA1C

PPV, positive predictive value; NPV, negative predictive value; AUC, area under the curve; CI, confidence interval

^a^Validated model

## Discussion

The present study describes the use of general clinical characteristics and biochemical indicators to establish a model for the diagnosis of insulinoma. Compared with the 72-h fasting test, our model is simpler and safer to diagnose insulinoma in hyperinsulinemic patients. Because the 72-h experiment brings patients a huge risk of hypoglycemia, increases the incidence of medical errors, and patients may not tolerate the experimental process and give up diagnosis [9].

Insulinoma can cause severe metabolic disorders. Long-term hypoglycemia can cause irreversible damage to nerve tissue, and even endanger the life of the patient in severe cases [17]. Therefore, early diagnosis and treatment are required. Insulinomas can be diagnosed qualitatively or based on localized factors [18]. Qualitative diagnosis mainly relies on clinical manifestations and laboratory tests. At present, the 72-h fasting test is the standard method for the qualitative diagnosis of insulinoma [6, 19–21]. An insulin-to-glucose ratio > 0.3 at the onset of hypoglycemia may provide a basis for diagnosing insulinoma. Many patients refuse to take the 72-h fasting test, due to its being a painful experience and its accompanying risk of hypoglycemia. A retrospective analysis of the results in 69 patients with confirmed insulinoma showed that 20 (29.0%) had negative results on 72-h fasting tests [22], indicating that negative results on 72-h fasting tests cannot completely rule out a diagnosis of insulinoma [23]. Insulinomas can also be diagnosed by imaging modalities, including ultrasound, CT, MRI, and endoscopic ultrasound (EUS), although the positivity rates of these noninvasive examinations are not high, with preoperative CT and MRI having sensitivity rates of 72% and 75%, respectively [24]. EUS, another minimally invasive method, was found to have a sensitivity of 94% [25], although its sensitivity was largely dependent on the operator’s technique and experience. The sensitivities of EUS in detecting insulinomas in the head and body of the pancreas were high, at 95% and 98%, respectively [26], whereas its sensitivity in detecting lesions in the pancreatic tail was much lower, ranging from 37 to 50% [27, 28]. Of the 37 patients pathologically diagnosed with insulinoma in the present study, 32 (86.5%) underwent EUS, with all 32 having space-occupying lesions, of minimum diameter 5.6 mm, in the tail of pancreas. Four false-negative cases were included in our model, two in the head of the pancreas and another two in the tail, and all tumors are smaller than 1 cm. These findings suggested that application of this model yielded errors in patients with atypical clinical symptoms due to the small size of these tumors. Other methods, such as PET-CT and GLP-1 receptor imaging, have been shown superior to MRI and CT in diagnosing insulinomas. However, their clinical application is limited due to the high costs of examinations.

Weight gain is a significant manifestation of insulinoma. Due to the frequent occurrence of hypoglycemia symptoms, patients can relieve symptoms such as palpitation, tremor, and dizziness through eating [29]. According to a retrospective study, 72% of patients with insulinoma have gained weight [15]. In our study, the BMI of insulinoma patients was in the overweight range, while the BMI of the control group was within the normal range. Among the biochemical indicators, the HbA1c of patients in the insulinoma group was significantly lower than that in the control group, which suggests that the plasma glucose of patients with insulinoma has been low for a long time. Combining plasma glucose, insulin and C-peptide, insulinoma patients have higher fasting insulin and fasting C-peptide than the control group, indicating that insulinoma patients are more likely to have fasting hypoglycemia symptoms. After taking 75 g anhydrous glucose powder, the indicators of insulinoma group, including 1-h plasma glucose and 2-h plasma glucose, are lower than the control group. Considering that insulinoma patients release a large amount of insulin, the improvement of plasma glucose level after taking anhydrous glucose powder is still relatively slow. The effect of insulinoma on patients’ plasma glucose is a relatively long-lasting process, which is extremely harmful to the human body.

Insulinomas are relatively rare and differ in clinical symptoms, making this condition easy to miss and misdiagnose [30–32]. A clear diagnosis of insulinoma is a prerequisite for standardized treatment. Fajans’ and Turner’s indices are often used to evaluate the role of insulin release from pancreatic beta cells in regulating plasma glucose. Previous testing techniques were unable to distinguish between insulin and proinsulin, leading to errors in the results of these indices [33]. In addition, these indices lack research on insulinoma disease, thus, leading to imprecise in the diagnosis of insulinoma [16, 33]. Compared with these indices, our model was more accurate and more reliable. A similar model of diagnosing insulinoma includes fasting insulin, fasting C-peptide, 1-h C-peptide, and 2-h insulin concentrations [9]. The AUC of this model was 0.97, with a sensitivity of 86.5% and a specificity of 95.2%. That model also cannot be validated because of the lack of large-scale, multi-center clinical data to verify the reliability of the model. Incorporation of our data into this model yielded a sensitivity of 70.3%; a specificity of 65.9%; and an accuracy of 67.9%. This result is lower than what they got. Another study compared 82 insulinoma patients with 100 healthy subjects and constructed the FPG*HBA1C index [34]. Because this control group consisted of normal persons, the results may not be as accurate. Compared with FPG*HBC1A index, which used control groups consisting of normal healthy individuals, our model used a control group consisting of patients with hypoglycemia not caused by insulinoma, making our model more reliable. In addition, our model combined BMI with glucose-related indicators and used both single factor and multi-factor analysis to obtain the optimal formula: Logit p = 7.399 + (0.310 × BMI) − (1.851 × HbA1c) − (1.467 × 0-h plasma glucose) + (1.963 × 0-h C-peptide) − (0.612 × 1-h plasma glucose). These indicators were based on single factor analysis and multi-factor logistic regression analysis. The result showed that best cutoff value was − 0.17, when the result is greater than this value, it is considered that the hypoglycemic patient may have insulinoma disease. Therefore, our results show that our model outperforms the others. The data included in our model consisted of routine screening parameters for patients with hypoglycemia, providing this model high clinical feasibility and easy implementation.

## Conclusion

In this study, the insulinoma diagnostic model constructed with non-invasive indicators has good diagnostic value, which is of great significance to discover more patients with insulinoma. In order to improve the stability of this model, it needs to be verified in more insulinoma patients.

## Data Availability

The datasets generated during and/or analysed during the current study are available from the corresponding author on reasonable request.
